# Pre-industrial plague transmission is mediated by the synergistic effect of temperature and aridity index

**DOI:** 10.1186/s12879-018-3045-5

**Published:** 2018-03-20

**Authors:** Ricci P. H. Yue, Harry F. Lee

**Affiliations:** 1 Department of Geography, The University of Hong Kong, Pok Fu Lam, Hong Kong; 2 International Center for China Development Studies, The University of Hong Kong, Pok Fu Lam, Hong Kong

**Keywords:** Plague, climate change, synergistic effect, Europe, *Yersinia pestis*

## Abstract

**Background:**

Although the linkage between climate change and plague transmission has been proposed in previous studies, the dominant approach has been to address the linkage with traditional statistical methods, while the possible non-linearity, non-stationarity and low frequency domain of the linkage has not been fully considered. We seek to address the above issue by investigating plague transmission in pre-industrial Europe (AD1347–1760) at both continental and country levels.

**Methods:**

We apply Granger Causality Analysis to identify the casual relationship between climatic variables and plague outbreaks. We then apply Wavelet Analysis to explore the non-linear and non-stationary association between climate change and plague outbreaks.

**Results:**

Our results show that 5-year lagged temperature and aridity index are the significant determinants of plague outbreaks in pre-industrial Europe. At the multi-decadal time scale, there are more frequent plague outbreaks in a cold and arid climate. The synergy of temperature and aridity index, rather than their individual effect, is more imperative in driving plague outbreaks, which is valid at both the continental and country levels.

**Conclusions:**

Plague outbreaks come after cold and dry spells. The multi-decadal climate variability is imperative in driving the cycles of plague outbreaks in pre-industrial Europe. The lagged and multi-decadal effect of climate change on plague outbreaks may be attributable to the complexity of ecological, social, or climate systems, through which climate exerts its influence on plague dynamics. These findings may contribute to improve our understanding of the epidemiology of plague and other rodent-borne or flea-borne infectious diseases in human history.

**Electronic supplementary material:**

The online version of this article (10.1186/s12879-018-3045-5) contains supplementary material, which is available to authorized users.

## Background

Although significant medical advancements in recent decades have lowered infectious disease mortality from 16 million to 15 million in 1990–2010 [1], there is no locale in the world that can classify infectious disease as a non-existent risk to mankind. One upcoming challenge originates from the effect of increasing temperature associated with climate change in facilitating the transmission of infectious diseases and enlarging the distribution of certain vector-borne diseases [2]. The effects of climate change on infectious disease are materialized through a set of complex pathways that involve various parts of nature, making the connection between climate change and infectious diseases difficult to trace. Historical evidence suggests that latitudinal, altitudinal, seasonal, and inter-annual associations between climate and disease superimpose on each other to affect infectious disease in a nonlinear manner [3, 4]. The connection between environmental and epidemiological dynamics is typically non-stationary [3], partially owing to the fact that epidemiological time series change along with surveillance systems and advancements in medication. Furthermore, the impact of vaccination and human intervention on disease outbreaks varies across different places and in different periods [5], which further complicates the study of climate change impact on epidemic outbreaks.

Despite of the fact that the non-linear and non-stationary nature of the connection between climate change and various types of infectious diseases has been highlighted in previous studies [6–10], the non-linearity and non-stationarity of the climate-plague nexus has rarely been systematically addressed, with only a few exceptions, such as the study of Kreppel et al. [11] on modern Madagascar or the work of Xu et al. [12] on the 19th and twentieth century China. The study time span of the climate-plague nexus is only extended back to AD1850, which is too short to reveal the evolution of the nexus in major climatic epochs such as the Medieval Warm Period (AD1000–1300) and the Little Ice Age (AD1400–1900). Currently, the effect of prolonged cooling on plague transmission remains unknown.

Plague is an infectious disease caused by the bacteria *Yersinia pestis*, which is transmitted to humans through rodent fleas. In many parts of the world, plague remains a major threat, particularly in Africa, where both the number of cases and the number of countries infected showed an increasing trend over the past decades [13]. According to a recent review, the known natural foci are evident in Africa (Mainly in Madagascar, Demographic Republic of Congo and Eastern Africa) and the Middle East (Central Asia region) [14]. As it is nearly impossible to design laboratory experiments to study plague transmission in human societies, plague transmission has been mainly examined using a retrospective approach [15, 16]. Under this approach, scholars employ historical records to reveal the major longitudinal change of plague frequency and reflect the characteristics of the plague-environment relationship. However, except for the influence of climate as afore-stated, the transmission of the disease is closely related to rodent ecology [17–21], flea ecology [22, 23], societal factors [24] and other environmental conditions [15, 25]. Given the complexity of plague transmission, the influence of climatic forcing on plague transmission is unlikely to be linear and stationary.

This study seeks to provide an empirical examination of the non-linear and non-stationary influence of climatic change on plague outbreaks in Europe in AD1347–1760. The casual relationship of climate-plague nexus is determined first. Then, the non-linearity and the non-stationarity of the nexus are further explored. The results presented in this study may be helpful in comprehending the epidemiology of rodent-borne and flea-borne infectious diseases in human history.

## Methods

### Study area and study period

Our study focuses on historical Europe after the outbreak of the Black Death (AD1347) until the start of the Industrial Revolution (AD1760). In October 1347, plague first appeared in Europe at the port of Messina in Sicily [26]. The disease spread through Europe during the following years and made its name as the most notorious pandemic in history. Hence, AD1347 is set as the starting year of our study. The Industrial Revolution started around AD1760. Since then, humans have entered into a new era of sanitation, transportation, and technology [27]. Therefore, we cut off our study period at the year AD1760 to avoid the disparity of plague dynamics between the pre-industrial and industrial eras.

**Fig. 1:**
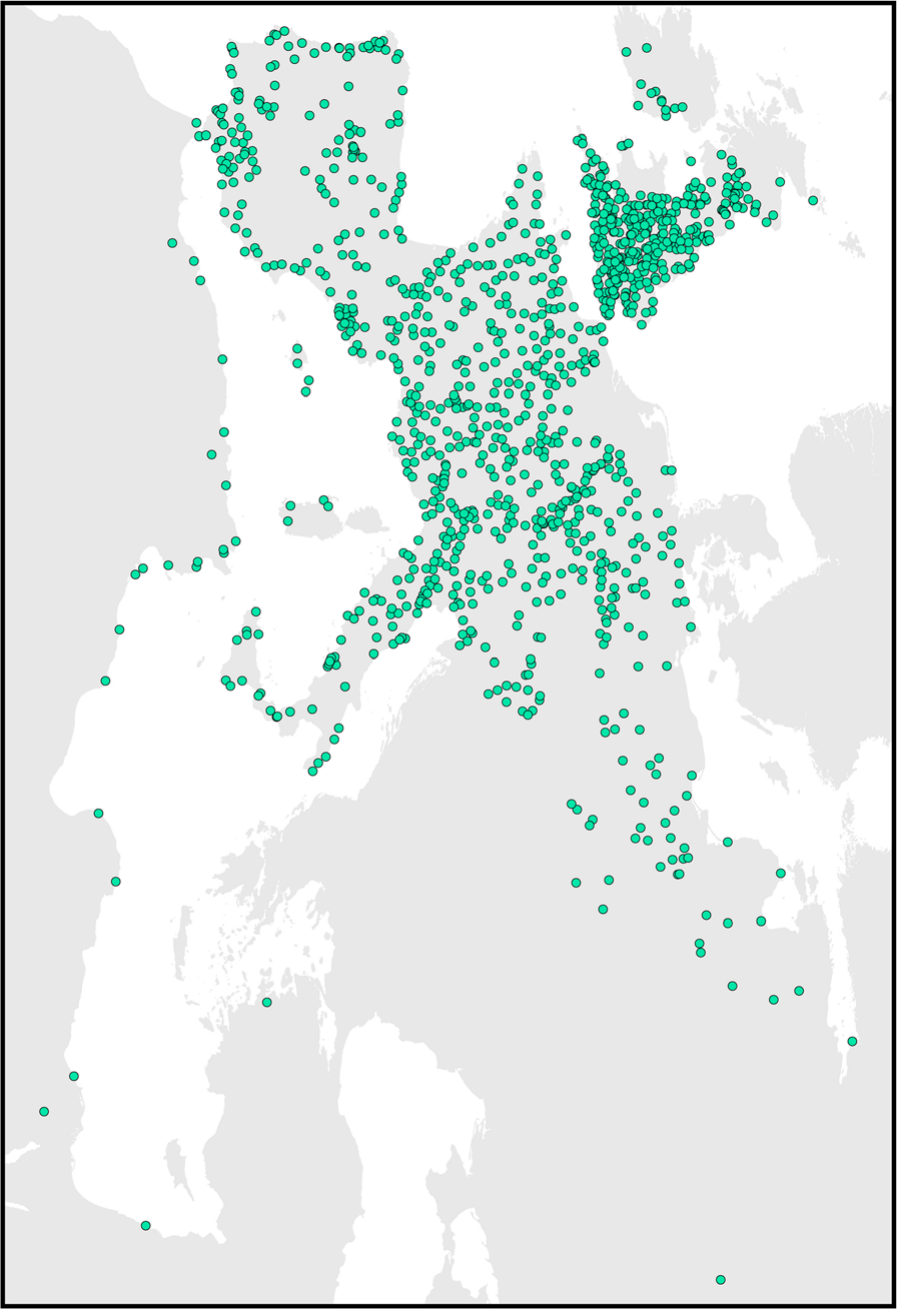
Map of Europe and North Africa with the indication of 6656 plague outbreak incidents in AD1347–1760. The dot size corresponds to the number of plague outbreak incidents at different locations

Our study area covers the entire European continent, as well as the northernmost part of Africa (Fig. 1) as suggested by Büntgen et al. [28], which takes into account the influence of the Africa-Europe connection on plague transmission during the time. In addition, five countries in Europe that were frequently affected by plague in history are singled out to further explore the effect of climate change on plague outbreaks at lower geographical levels (i.e., country). 88.0% of the total plague outbreak incidents in the dataset we employed are recorded in these five countries. The countries are delineated according to current administrative boundaries. Our aridity index and plague data are aggregated accordingly.

### Climate data

**Fig. 2:**
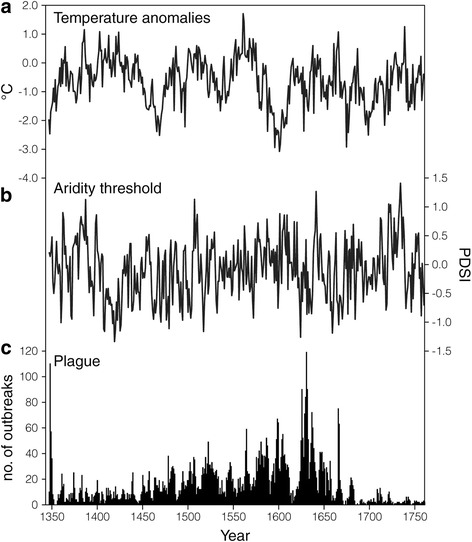
Climatic change and plague outbreak in Europe in AD1347–1760. **a** Temperature anomalies (°C), (**b**) PDSI, which is the proxy of aridity index, and **c** plague outbreak incidents

Climate in this study stands for the observation of climatic variables: temperature and aridity index. Our temperature data is extracted from the reconstructed temperature dataset compiled by Büntgen et al. [29] (Fig. 2a). In the dataset, the temperature anomaly series is derived with respect to the period of AD1901–2000. A total of 1547 tree ring chronologies are used for the annual temperature reconstruction. Such a large sample size ensures the reliability of the resultant temperature reconstruction, and the data has been frequently used in other historical studies of Europe [30–33].

Our aridity index is represented by the Palmer Drought Severity Index (PDSI). PDSI is chosen as the indicator of aridity index because it takes into account both precipitation and soil moisture [34] and it represents the severity of drought in a given period. PDSI is deemed to be a closely relevant variable to account for the response of biological beings to the change of the hydrological condition of the environment [35]. The PDSI data is obtained from the *Old World Drought Atlas* (OWDA) by Cook et al. [36] (Fig. 2b). The OWDA is a self-calibrated PDSI database at annual time step based on uniform tree ring chronology across the entire continent of Europe, with its negative value representing dry conditions and vice versa. For those studies pertinent to ecological systems, the dataset assembles not only the record from living trees, but also from an exceptional amount of historical, archaeological, and sub-fossil tree ring chronologies [36]. To transform ODWA into a time series, we average the PDSI value of each year separately. Country scale data is aggregated based on current country boundary. This adopted PDSI dataset can clearly reveal the wetness/dryness pattern of historical Europe, which has been adopted in other studies as well [37, 38]. For the description of precipitation data that we use for validity check, please refer to Additional file 1: Figures S1 and SI text.

### Plague data

Plague outbreak data in Europe in AD1347–1760 is extracted from the geo-referenced historical plague database compiled by Büntgen et al. [28] (Fig. 2c). There are a total of 6656 plague outbreak incidences documented in the inventory in our study period. Each plague outbreak incident includes the corresponding coordinates and occurrence year. They are entered to the dataset, and we transform them into a time series in annual resolution. Since the inventory does not provide the exact plague outbreak date and the duration of each outbreak, those outbreaks happening at the same location in the same year are counted as one plague outbreak incident [25, 39]. Plague data of the same outbreak year are aggregated together to form a time series. Our plague dataset has also been employed in recent studies on historical plague dynamics [16, 40], but the related researches focus on the influence of transportation routes on plague transmission.

### Granger causality analysis

Granger Causality Analysis (GCA) is an effective method to build a causal relationship between two time series [41]. Statistically, GCA provides the probabilistic evidence to tell if one variable ‘precedes its effect’ on the other in time, and whether the causal time series contains significant information for the forecast of the resulting time series [42]. Before applying GCA, the Augmented Dickey-Fuller test (ADF) is applied to check for the stationarity of data. If necessary, the time series are subjected to the corresponding level of differencing. The lag applied in the regression is determined by Akaike’s Information Criterion (AIC) [43].

### Wavelet analysis

Wavelet analysis is described as the microscope of signals [44], as it identifies the statistical relationship between signals and the dynamics of periodicity over time [45, 46]. It is well suited for the investigation and exploration of non-stationary relationships between time-series. The method decomposes signal in the time series into time-frequency space with multiple temporal dimensions [47]. Further, this decomposition allows the analysis to explore the dominant modes of co-variance and possible distribution of response to the predictor variables in terms of time and frequency [48].

In this study, we apply four kinds of wavelet analysis: cross wavelet transform (XWT), wavelet transform coherence (WTC), partial wavelet coherence (PWC), and multiple wavelet coherence (MWC). Before carrying out the analysis, we need to choose the mother wavelet as the basic function of time series transformation. Morlet wavelet is chosen since it is one of the most efficient means to decompose and analyze the signals [49]. For the variables of interest used in WTC, PWC and MWC, a normality test is performed to ensure the time series are not too far from Gaussian distribution [46]. If necessary, variables are normalized by Johnson transformation [50].

XWT offers a way to characterize the interaction between the wavelet transform of two individual time series by analyzing their common power and phase lag as a function of both time and frequency [51, 52]. The resulting plot will expose information of phase relationship and areas of high common power in terms of time-frequency domain [46]. WTC measures the local coherence of two time series via Monte Carlo methods [46]. High coherence in WTC means that plague co-varies with the investigated climatic index at a particular time-frequency domain [9]. Briefly, XWT provides more information on phase relationship and degree of common power, while WTC finds significant localized correlation coefficient [46]. Our XWT and WTC packages are provided by Grinsted et al. [46]. Wavelet transform of the time series employed in this study can be found in Additional file 1: Figure S2.

PWC is similar to partial correlation but in the form of wavelet analysis [53]. It helps to eliminate the influence of other variables in the original WTC. Using this study as an example, PWC helps us find the independent coherence of the time series of temperature on the time series of plague after eliminating the effect of aridity index. In this case, since the influence of aridity index is controlled, we can access whether the variance in temperature alone has any effect on plague. It can also provide information of whether variability in plague dynamic is forced by temperature via the alternation of aridity condition.

MWC is, on the other hand, designed to seek the WTC of multiple independents on a dependent [54]. The resulting wavelet coherence is the combination of explanatory power of X_1_ and X_2_ at a given time and frequency. In our study, it refers to how climate (a combination of temperature and aridity index) influences plague. Consequently, MWC can reveal how climatic indices work together in shaping the variation in plague outbreaks in temporal domain. Our PWC and MWC packages are provided by Ng and Chan [55].

### Procedures

In the first part of the assessment, we adopt GCA to validate the casual linkage between three pairs of time series: (1) temperature and plague outbreaks; (2) aridity index and plague outbreaks; and (3) precipitation and plague outbreaks. Before running GCA, ADF test is applied for checking the stationarity of all variables and an optimum lag length for GCA is obtained from AIC. In this study, GCA is used for the preliminary inspection of the climatic variables which are significant in driving plague dynamics in preindustrial Europe and to provide information for the lagged relationship.

A more systematic assessment of the climate-plague relationship is done by wavelet analysis. First, we apply XWT and WTC analysis between temperature and plague outbreaks and between aridity index and plague outbreaks to offer a holistic assessment of the climate-plague nexus on a macro scale. After that, MWC and PWC are employed to explore the synergistic effect of temperature and aridity index on plague outbreaks, the topic that has not been fully addressed in previous studies. Also, we repeat the MWC and PWC analyses on five countries in Europe that were frequently attacked by plague in history to further explore the synergistic effect of temperature and aridity index on plague outbreak at lower geographical levels (i.e., country). We also provide MWC result for precipitation-plague association (Additional file 1: Figure S3) and repeat the wavelet analysis with a plague time series which considers the influence of population growth in the corresponding spatial unit (Additional file 1: Figures. S4–S6).

## Results

### Climate-plague nexus in pre-industrial Europe in general

Based on the result of ADF test, zero differencing should be carried out for the dataset tested in GCA (Additional file 1: Table S1). The lag years for GCA are obtained from the AIC as presented in Additional file 1: Table S2. The AIC lag indicates that plague outbreaks lag behind the change of temperature and aridity index for five years. Results from GCA support the claim that both temperature and aridity index *Granger-cause* plague outbreaks while precipitation does not (Table 1).

**Table 1:** Results of GCA

Null hypothesis	*F*	p
TEMPERATURE does not *Granger-cause* plague outbreak	2.216	0.050
ARIDITY INDEX does not *Granger-cause* plague outbreak	3.505	0.004
PRECIPITATION does not *Granger-cause* plague outbreak	1.964	0.118

**Fig. 3:**
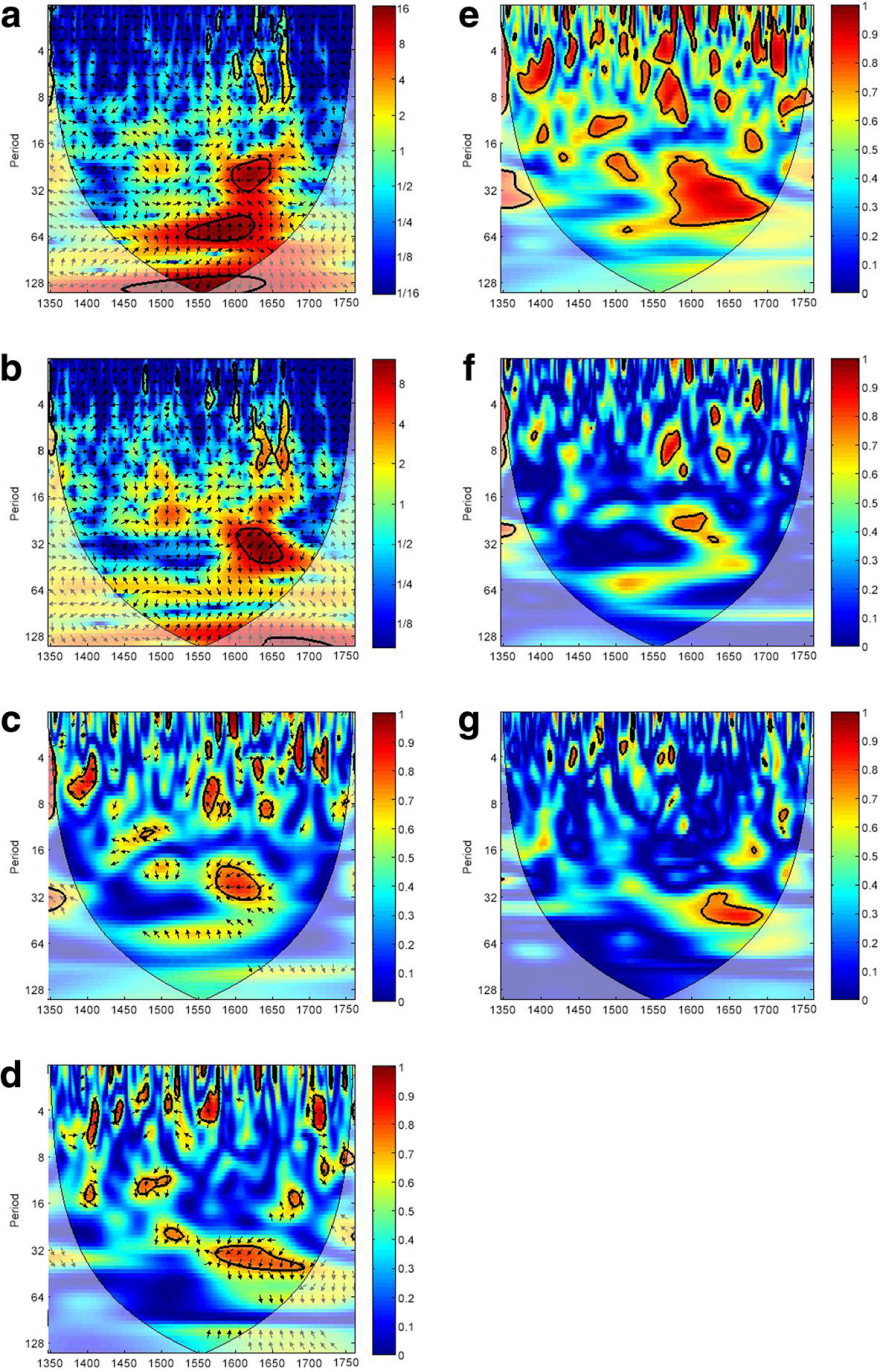
Wavelet analyses of the phase and frequency of the climate-plague nexus in Europe in AD1347–1760. **a** XWT analysis of temperature and plague. **b** XWT analysis of aridity index and plague. **c** WTC analysis of temperature and plague. **d** WTC analysis of aridity index and plague. **e** MWC analysis of temperature, aridity index, and plague. **f** PWC analysis of temperature and plague, with the effect of aridity index controlled. **g** PWC analysis of aridity index and plague, with the effect of temperature controlled. The color code for the spectrum refers to the significance of the relationship, ranging from dark red (high values) to dark blue (low values). Region with significant coherency (*p* < 0.05) against red noise is indicated by the black contour line. In the graph, the cone of influence indicates regions not influenced by edge effect

XWT and WTC results of climate-plague nexus in pre-industrial Europe are shown in Fig. 3. The yellow-red region indicates a statistically significant high level of common power and coherence. Temperature and plague dynamics exhibits a significant common power at the 16–32 year periodicities in AD1580–1650 (Fig. 3a and c). The XWT result also shows a consistent common power at the 32–64 year band in AD1540–1620. Within the significant region of coherence recorded in XWT and CWT, an anti-phase relationship is found, which indicates that when temperature drops, the number of plague outbreaks increases. We also analyze the influence of aridity index on plague transmission by XWT. Results show a region of high common power at the ~ 32 year band in AD1600–1670 (Fig. 3b). From CWT result, we see that there is a dominant coherence band at the ~ 32 year periodicities in AD1570–1670 (Fig. 3d). The associated phase relationship is close to anti-phase, implying that the number of plague incidents increases along with drought conditions. As a whole, the ~ 32 year coherence band in AD1600–1650 is common in both temperature-plague and aridity-plague association. This period is coincident with the coldest period in Europe observed over the last two millennia [29, 56].

### Synergistic effect of temperature and aridity index on plague transmission

Our WTC results reveal that both temperature and aridity index are significant drivers of plague variability in pre-industrial Europe. Yet, instead of working individually, temperature and aridity index may interact in affecting plague dynamics, and such interaction effect cannot be addressed by WTC. Therefore, we apply MWC to investigate the possible synergistic effect of temperature and aridity index on plague transmission. The result is presented in Fig. 3e, which indicates that the combined effect of temperature and aridity index has a very strong influence on plague outbreaks. In AD1500–1530, there is a strong coherence between the temperature-aridity synthesis and plague transmission of the 8–16 year and 16–32 year periodicities. The coherence becomes less significant in AD1530–1560, while a strong and consistently significant coherence of the 16–64 year periodicities emerges in AD1560–1700. Although this significant region of coherence can also be seen on the coherent spectra in the WTC result (Figs. 3c and 3d), the one in the MWC result is much bigger (Fig. 3c). This suggests that the combined effect of temperature and aridity index on plague transmission is larger than the individual impact of each of the climatic variables.

### Individual effect of temperature and aridity index on plague transmission

When verifying the relationship between independent variable and targeted dependent variable by using WTC, the ‘stand alone’ influence of the independent variable on the dependent variable cannot be shown. The problem can be solved only if the effect of possible common dependence is eliminated. Therefore, we apply PWC to check the individual impact of temperature and aridity index on European plague outbreaks. For the independent influence of temperature on plague dynamics, the covariance is largely diminished after controlling the effect of aridity index (Fig. 3f). Only a small coherence region at the 16–32 year periodicities in AD1500–1540 and AD1570–1630 is observed. Alternatively, when we examine the influence of aridity index on plague by controlling the effect of temperature, the result only supports a minor covariance of ~ 32 year periodicities in AD1610–1680 (Fig. 3g). The above results suggest that neither temperature nor aridity index is strongly associated with plague transmission in pre-industrial Europe. Furthermore, the significant climate-plague association is largely confined to certain periods. Comparing the result of PWC (Figs. 3f and 3g) with that of WTC (Figs. 3c and 3d), it is apparent that the size of coherent region shrinks and the continuity of band reduces in the former.

Another point to note is that when comparing the result of MWC (Fig. 3e) with that of PWC (Figs. 3f and 3g), the size of the significant region is much smaller in the latter. From the comparison of figures, we can see that such reduction of significant association is not only notable in low-frequency (16–64 year) and middle-frequency (8–16 year) coherence bands, but also in the high-frequency (2–8 year) coherence band, even though such coherence is not consistent and continuous. On the other hand, coherence that is significant in the PWC analysis (64 year periodicities in AD1640–1680) becomes strongly significant in MWC analysis.

The above results come with two observations. First, the synergistic effect of temperature and aridity index significantly associates with plague transmission at the multi-decadal time scale. Second, the ‘stand alone’ influence of temperature and aridity index is not persuasive in explaining the dynamics of plague outbreaks, implying that in a theoretical situation, when temperature (aridity index) change is controlled, the association between drought (cooling) and plague outbreaks will largely diminish.

### Synergistic effect of temperature and aridity index on plague transmission at the country level

Although the synergistic effect of temperature and aridity index on plague transmission at the continental level has been demonstrated in our MWC and PWC results (Figs. 3e–3g), such relationship should not be downscaled right away. This is because climate-man interaction at the continental scale may not be identical to the one at lower geographic levels [39, 57, 58]. Owing to this concern, we also conduct MWC and PWC analysis in five of the countries in Europe (UK, France, Germany, Italy, and Spain) to check whether the synergistic effect of temperature and aridity index on plague dynamics is still significant at the country level.

**Fig. 4:**
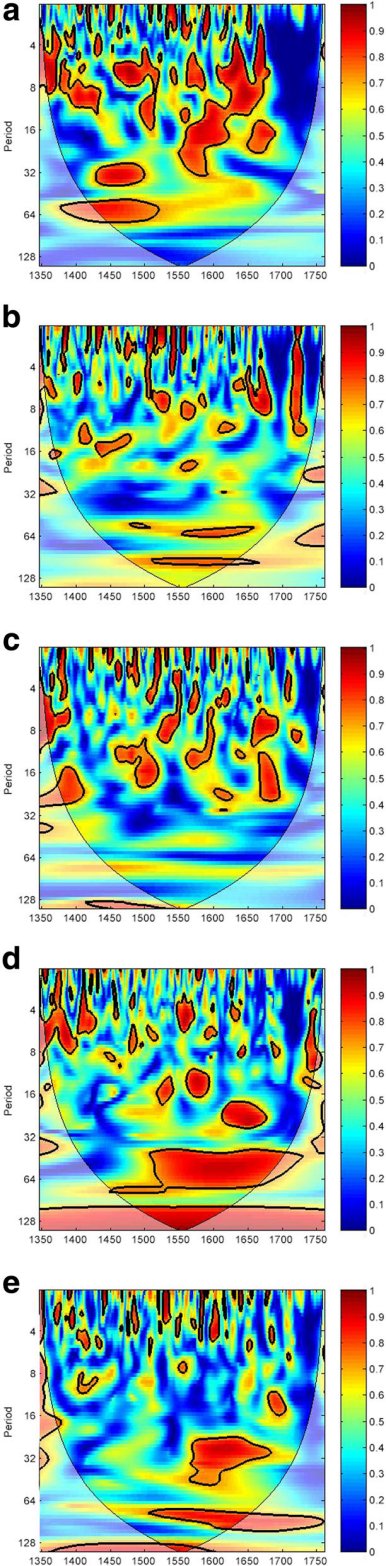
MWC analysis of the climate-plague nexus in the five countries in Western Europe in AD1347–1760. **a** UK, (**b**) France, (**c**) Germany, (**d**) Italy, and (**e**) Spain. The color code for the spectrum refers to the significance of the relationship, ranging from dark red (high values) to dark blue (low values). Region with significant coherency (*p* < 0.05) against red noise is indicated by the black contour line. In the graph, the cone of influence indicates regions not influenced by edge effect

Our MWC results are presented in Fig. 4. The coherence between the temperature-aridity synthesis and plague transmission in our chosen countries remains significant, except in Germany. A stable and significant coherence band of ~ 32 year periodicities and ~ 64 year periodicities appeared in the UK in AD1430–1510 and AD1370–1530, respectively (Fig. 4a). There is also scattered coherence at the 16–32 year periodicities in the UK in AD1560–1700. The same periodicities also appear in a similar period in Italy (AD1620–1690, Fig. 4d) and Spain (AD1570–1690, Fig. 4e). The most notable and consistent result in the country-scale wavelet results all appear at the multi-decadal time scale. In Italy, a very consistent coherence band of 32–64 year periodicities can be spotted in AD1515–1740. France has a consistent coherence band of 64 year periodicities in AD1560–1665 (Fig. 4b). In addition, the coherence band of 64–128 year periodicities is dominant in France (AD1515–1665), Italy (AD1347–1760) and Spain (AD1510–1760). Briefly, the above results also imply that plague dynamics are associated with the combined effect of temperature and aridity index mainly at the multi-decadal time scale.

**Fig. 5:**
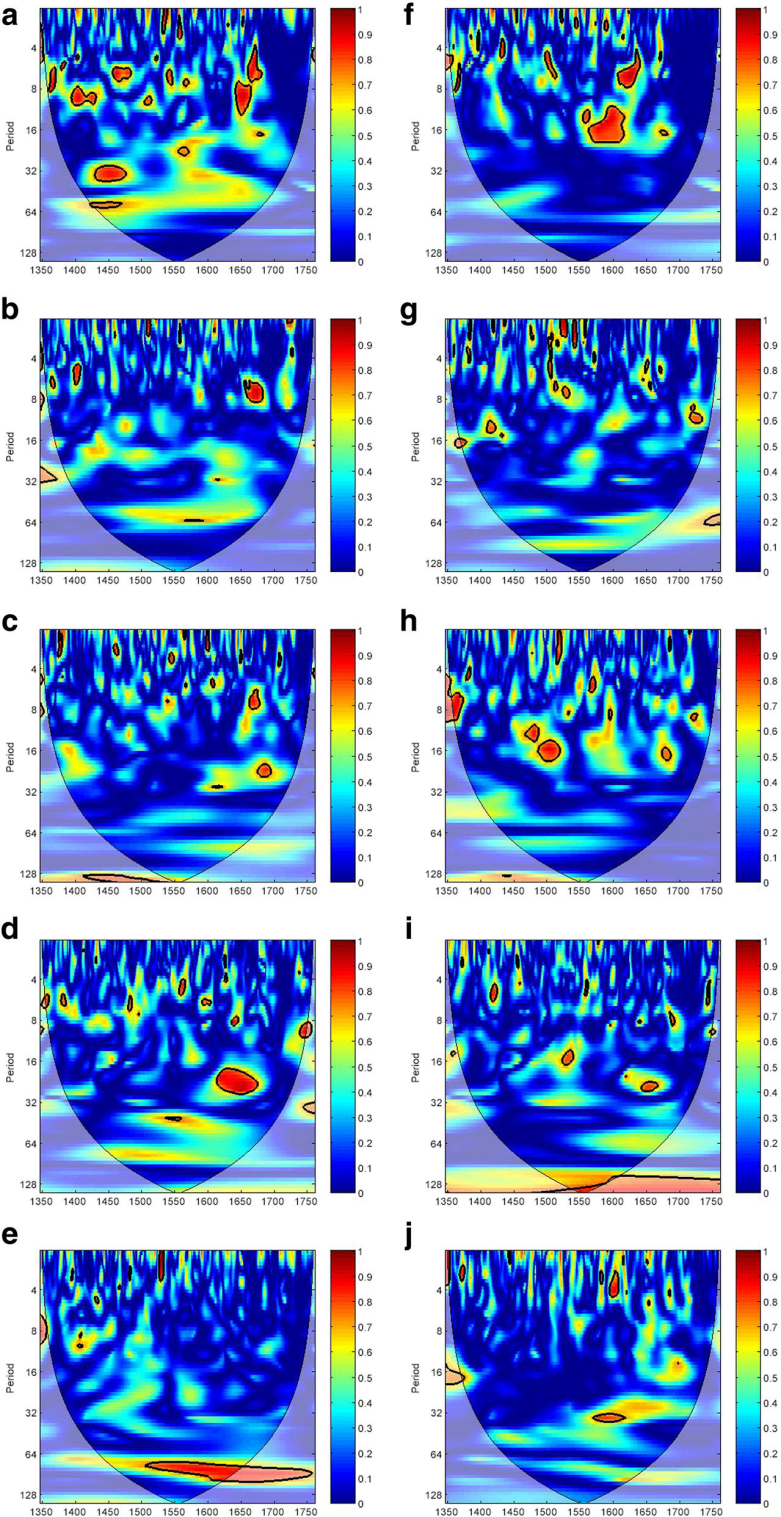
PWC analysis of the climate-plague nexus in the five countries in Western Europe in AD1347–1760. Panels in the left column are the PWC analysis of temperature and plague, with the effect of aridity index controlled: (**a**) UK, (**b**) France, (**c**) Germany, (**d**) Italy, and (**e**) Spain. Panels in the right column are the PWC analysis of aridity index and plague, with the effect of temperature controlled: (**f**) UK, (**g**) France, (**h**) Germany, (**i**) Italy, and (**j**) Spain. The color code for the spectrum refers to the significance of the relationship, ranging from dark red (high values) to dark blue (low values). Region with significant coherency (*p* < 0.05) against red noise is indicated by the black contour line. In the graph, the cone of influence indicates regions not influenced by edge effect

Moving to the PWC results of temperature and aridity index with plague transmission at the country level (Fig. 5), they are somewhat consistent in comparison with that for the entire European continent. After controlling the effect of aridity index, we cannot spot any consistent coherency between temperature and plague in the UK, France, and Germany (Figs. 5a–5c). But there is a small extent of temperature-plague coherency of the 16–32 year periodicities in Italy in AD1615–1680 (Fig. 5d). Spain is the only country having a significantly consistent temperature-plague coherency of 64–128 year periodicities in AD1500–1760 (Fig. 5e). Regarding the individual effect of aridity index on plague, after controlling the effect of temperature, no significant coherence band can be found in the five countries investigated (Figs. 5f–5j). Again, the PWC result also points to the synergistic effect of temperature and aridity index on plague outbreaks as described in the previous sections. To a large extent, temperature and aridity index have an effect on plague dynamic only if they work together. Such synergy is not only valid at the continental level, but also valid at the country level.

## Discussion

Climate change has been widely acknowledged as a key factor in determining the spatio-temporal dynamics of various infectious diseases [59, 60], and the epidemiological influence of climate is proven to be mainly non-linear and non-stationary [6, 8, 11, 61]. The climatic dependency of plague transmission has also been identified in Madagascar, Central Asia and China [11, 12, 62], despite the role of different climatic variables in different spatio-temporal contexts. We take a step forward to explore the climatic dependence of plague dynamics in pre-industrial Europe and reveal a lagged and non-stationary relationship between climate change and plague outbreaks.

Our GCA results show that temperature and aridity index have a causal relationship with plague outbreaks, while the lag between climate change and plague outbreaks is most significant at a 5-year interval (Additional file 1: Tables S2 and Fig. 2). Wavelet analysis further confirms the role of temperature and aridity index in causing plague transmission at the continental and country levels. Both climatic variables have an anti-phase multi-decadal coherence with plague outbreaks (i.e., plague outbreaks associate with cold and dry spells). The discovery of negative association between temperature and plague dynamics is opposite to some previous studies [20, 62]. Yet, as highlighted by Xu et al. [12], climate-plague relationship can experience an opposite response in different spatio-temporal settings. Although both temperature and aridity index are the significant determinants of plague outbreaks, their synergy is more important than their individual effect in driving plague transmission (Fig. 3). The climate-plague nexus is characterized by the multi-decadal (16–64 year) periodic mode in AD1347–1760, except in Germany (Fig. 4). In addition, the coherence of 64–128 year periodicities is found in France, Italy, and Spain in AD1347–1760 (Fig. 4). Such lagged and multi-decadal relationship is different from the seasonal to annual covariance between climate and plague [11] or the immediate plague effect of climatic variations [63].

There are a few compatible plausible explanations to our findings. The first explanation addresses the behavioral plasticity of the ecosystems, through which the effect of climatic variations on plague dynamics is operationalized. Ecosystems are flexible, especially for rodents [64]. Although the life cycle of rodents is short, they are very adaptable to short-term environmental variation in terms of temperature, humidity, and food supply due to their capability in behavioral adjustments [65]. On one hand, these adjustments regulate rodent population [66]. On the other hand, the impacts brought by short-term (i.e., annual to multi-annual) climate oscillations on rodents are also buffered by such behavioral plasticity. However, such mechanism is not capable of coping with the ecological stress brought by long-term (i.e., multi-decadal or longer) deteriorations of temperature and aridity index. According to our results for the climate-plague nexus (Table 1), the flexibility in ecosystems may allow rodents to regulate their own dynamics when either the temperature or the aridity condition deteriorates. Yet, when multi-decadal cold and dry spells are synchronous and overshoot the flexibility of ecosystem, the behavioral plasticity can only delay, rather than dissolve, the synergistic effect of temperature and aridity index in driving plague outbreaks.

The biologically plausible mechanism should not only be built on rodent vectors, but also be built on flea vectors. Archaeological evidence suggests that black rats were largely absent in large areas of Northern Europe in the Black Death era due to harsh climatic conditions [67] and hence, unlikely to be responsible for any plague transmission during that period. On the other hand, evidence from Madagascar shows that the major flea/plague vectors are more adant in colder highland areas at over 800 m a.s.l. [22], formulating the highly geographically restricted plague endemic regions in highlands [68]. Fleas in Europe may behave similarly and become more abundant in cold and dry periods, which facilities the transmission of plague. Still, the relative importance between rodents and fleas in plague outbreaks throughout European history should be further explored. Indeed, the synergistic effect of temperature and aridity index has been demonstrated in other of ecological studies [69, 70], but this is the first time it has been discovered in plague dynamics.

Also, it may be possible that the effect of climate oscillations is mediated by other parts of social systems such as wars, famines, migrations and so on. Existing literature has demonstrated the profound impact of climate deterioration on historical agrarian economy at the multi-decadal time scale [31, 71–73]. Socio-economic downturn brought by multi-decadal climate change would associate with unaffordable food prices and the deterioration of social buffers [74], which would further lead to widespread malnutrition and epidemic outbreaks [39]. In situations of economic hardship, public health and hygiene provision is hampered, which exacerbates disease outbreaks [75].

Another notable explanation to our findings is that there is a larger forcing behind the climatic proxies applied in this study. While it is possible, both temperature and aridity conditions are controlled by multi-decadal climatic phenomena, and therefore the climate-plague coherence always exist at the multi-decadal time scale. As climatic variables in smaller scales are manipulated by multiple larger climatic forcings, our observation of their effect on plague dynamics through temperature and aridity index will certainly be obscured. In fact, the effect of large-scale climatic forcings such as El Niño Southern Oscillation and Pacific Decadal Oscillation on plague dynamics has been highlighted in previous studies [11, 76, 77]. The observed climate-plague coherence may simply reflect the effect of some large-scale climatic forcings on plague outbreaks in Europe. For instance, large scale climatic influences such as North Atlantic Oscillation and Atlantic Multidecadal Oscillation have a profound impact on climate change and consequently on human societies in European history [57, 58, 78]. We recommend further investigation to clarify their role.

This is not the first time the association between temperature and epidemic outbreaks at the multi-decadal temporal scale has been identified [20, 71, 72, 79–81]. Previous work has also demonstrated a similar idea that temperature is more imperative in explaining the pattern of epidemics [32]. However, the effect of aridity index in mediating the temperature-epidemics nexus has been overlooked in the abovementioned studies. Conventional statistical methods such as correlation and multiple regressions may fail to reveal the non-stationary and non-linear association between climatic variations and plague dynamics. With the quantitative link between temperature, aridity index and plague dynamics presented in this study, information for better understanding the climate-plague nexus in pre-industrial Europe on a longer time scale can be obtained.

Nevertheless, this study is limited by the resolution and quality of the data sources. Following the description of the digitalized dataset edited by Büntgen et al. [28], there is a lack of further classification to identify if the plague outbreak appears in the form of bubonic plague or pulmonary plague. Also, the original dataset employed by Büntgen et al. [28] has been recognized as being over-representative of Western Europe (Fig. 1) [82]. On the other hand, the statistical methods applied in this study may be subject to their own limitations. For instance, despite of the fact that wavelet analysis is a very powerful tool in decomposing time series to extract specific information in the time-frequency domain, the discontinuous coherence may be difficult to interpret in some occasions. While GCA is a probabilistic measurement of causal relationship, and wavelet analysis contains information about phase difference between time series and their coherence, detailed explanations for the associations are indistinguishable. Also, simple regression result suggests a weak but significant association between temperature and aridity index (Additional file 1: Table S3). Our proposed mechanism for the climate-plague nexus should be further verified in future study and any application of result should be dealt with care.

## Conclusion

This study focuses on the non-linearity and non-stationarity of the climate-plague nexus in Europe in AD1347–1760. Our GCA results show that both temperature and aridity index are significant in causing plague outbreaks in pre-industrial Europe, with a 5-year time lag. Our wavelet analysis results show that although temperature and aridity index exhibit a negative association with plague outbreaks, their synergy is more vital than their individual effect in driving the plague outbreaks. At the continental level, a significant coherency in 16–64 year periodicities between plague outbreaks and the synergy of temperature and aridity index is found. At the country level, the significant coherency between plague outbreaks and the synergy of temperature and aridity index is in 16–128 year periodicities. Such lagged and multi-decadal connection between climate change and plague outbreaks may be attributable to the complexity of ecological, social, or climate systems in operationalizing the climate-plague nexus. The above findings may offer new insight to the study of climate-epidemic nexus and help to explore how different environmental factors work together/separately in affecting human societies. Future research may leverage our results and further explore how the spatio-temporal dynamics of plague transmission is shaped by climate change.

## Additional file

Additional file 1: **Figure S1.** Time series of precipitation in Europe in AD1347–1760. **Figure S2.** Wavelet transform of the time series of (A) plague outbreak; (B) temperature; (C) aridity index; and (D) precipitation. **Figure S3.** MWC analysis of the synergistic effect of temperature and precipitation on plague dynamics in Europe. **Figure S4.** Wavelet analyses of the phase and frequency of the climate-plague nexus in Europe in AD1347–1760. With the time series of plague outbreak normalized by historical population figures from McEvedy and Jones [1]. (A) WTC analysis of temperature and plague. (B) WTC analysis of aridity index and plague. (C) MWC analysis of temperature, aridity index, and plague. (D) PWC analysis of temperature and plague, with the effect of aridity index controlled. (E) PWC analysis of aridity index and plague, with the effect of temperature controlled. The color code for the spectrum refers to the significance of the relationship, ranging from dark red (high values) to dark blue (low values). Region with significant coherency (*p* < 0.05) against red noise is indicated by the black contour line. In the graph, the cone of influence indicates regions not influenced by edge effect. **Figure S5.** MWC analysis of the climate-plague nexus in the five countries in Western Europe in AD1347–1760. With the time series of plague outbreak adjusted by historical population figures in the corresponding country from McEvedy and Jones [1]. (A) UK, (B) France, (C) Germany, (D) Italy, and (E) Spain. The color code for the spectrum refers to the significance of the relationship, ranging from dark red (high values) to dark blue (low values). Region with significant coherency (*p* < 0.05) against red noise is indicated by the black contour line. In the graph, the cone of influence indicates regions not influenced by edge effect. **Figure S6.** PWC analysis of the climate-plague nexus in the five countries in Western Europe in AD1347–1760. With the time series of plague outbreak adjusted by historical population figures in the corresponding country from McEvedy and Jones [1]. Panels in the left column are the PWC analysis of temperature and plague, with the effect of aridity index controlled: (A) UK, (B) France, (C) Germany, (D) Italy, and (E) Spain. Panels in the right column are the PWC analysis of aridity index and plague, with the effect of temperature controlled: (F) UK, (G) France, (H) Germany, (I) Italy, and (J) Spain. The color code for the spectrum refers to the significance of the relationship, ranging from dark red (high values) to dark blue (low values). Region with significant coherency (*p* < 0.05) against red noise is indicated by the black contour line. In the graph, the cone of influence indicates regions not influenced by edge effect. **Table S1.** Results of ADF test. **Table S2.** Difference level and AIC lag of casual linkages. **Table S3.** Simple regression result for temperature and aridity index association. (DOCX 4816 kb)

## Abbreviations

ADF: Augmented Dickey-Fuller
AIC: Akaike’s Information Criterion
GCA: Granger Causality Analysis
MWC: Multiple Wavelet Coherence
OWDA: Old World Drought Atlas
PDSI: Palmer Drought Severity Index
PWC: Partial Wavelet Coherence
WTC: Wavelet Transform Coherence
XWT: Cross Wavelet Transform
