# Supplementary material for: Pre-industrial plague transmission is mediated by the synergistic effect of temperature and aridity index
Source: BMC Infect Dis. 2018 Mar 20;18:134. doi: 10.1186/s12879-018-3045-5 (PMC5859406; doi:10.1186/s12879-018-3045-5)
Supplement: Supplementary file 1 — Figure S1. Time series of precipitation in Europe in AD1347–1760. Figure S2. Wavelet transform of the time series of (A) plague outbreak; (B) temperature; (C) aridity index; and (D) precipitation. Figure S3. MWC analysis of the synergistic effect of temperature and precipitation on plague dynamics in Europe. Figure S4. Wavelet analyses of the phase and frequency of the climate-plague nexus in Europe in AD1347–1760. With the time series of plague outbreak normalized by historical population figures from McEvedy and Jones [1]. (A) WTC analysis of temperature and plague. (B) WTC analysis of aridity index and plague. (C) MWC analysis of temperature, aridity index, and plague. (D) PWC analysis of temperature and plague, with the effect of aridity index controlled. (E) PWC analysis of aridity index and plague, with the effect of temperature controlled. The color code for the spectrum refers to the significance of the relationship, ranging from dark red (high values) to dark blue (low values). Region with significant coherency (p < 0.05) against red noise is indicated by the black contour line. In the graph, the cone of influence indicates regions not influenced by edge effect. Figure S5. MWC analysis of the climate-plague nexus in the five countries in Western Europe in AD1347–1760. With the time series of plague outbreak adjusted by historical population figures in the corresponding country from McEvedy and Jones [1]. (A) UK, (B) France, (C) Germany, (D) Italy, and (E) Spain. The color code for the spectrum refers to the significance of the relationship, ranging from dark red (high values) to dark blue (low values). Region with significant coherency (p < 0.05) against red noise is indicated by the black contour line. In the graph, the cone of influence indicates regions not influenced by edge effect. Figure S6. PWC analysis of the climate-plague nexus in the five countries in Western Europe in AD1347–1760. With the time series of plague outbreak adjusted by historical po [file 12879_2018_3045_MOESM1_ESM.docx]

**Plague dynamics is controlled by the synergistic effect of climatic indices**

**Table S1.** Results of Augmented Dickey-Fuller test

**Table S2.** Difference level and Akaike’s information criterion lag of casual linkages

**Table S3.** Simple regression result for temperature and aridity index association

**Figure S1.** Time series of precipitation, AD1347–AD1760

**Figure S2.** Wavelet transform of the time series of (A) plague outbreak; (B) temperature; (C) PDSI; and (D) precipitation.

**Figure S3.** Multiple wavelet coherence (MWC) analysis, which demonstrates the synergistic effect of temperature anomalies and precipitation on plague dynamics in Europe.

**Figure S4.** Wavelet analyses of the phase and frequency of the climate-plague nexus in Europe in AD1347–1760. With the time series of plague outbreak normalized by historical population figures from McEvedy and Jones [1]. (A) WTC analysis of temperature and plague. (B) WTC analysis of aridity index and plague. (C) MWC analysis of temperature, aridity index, and plague. (D) PWC analysis of temperature and plague, with the effect of aridity index controlled. (E) PWC analysis of aridity index and plague, with the effect of temperature controlled. The color code for the spectrum refers to the significance of the relationship, ranging from dark red (high values) to dark blue (low values). Region with significant coherency (p < 0.05) against red noise is indicated by the black contour line. In the graph, the cone of influence indicates regions not influenced by edge effect.

**Figure S5.** MWC analysis of the climate-plague nexus in the five countries in Western Europe in AD1347–1760. With the time series of plague outbreak adjusted by historical population figures in the corresponding country from McEvedy and Jones [1]. (A) UK, (B) France, (C) Germany, (D) Italy, and (E) Spain. The color code for the spectrum refers to the significance of the relationship, ranging from dark red (high values) to dark blue (low values). Region with significant coherency (p < 0.05) against red noise is indicated by the black contour line. In the graph, the cone of influence indicates regions not influenced by edge effect.

**Figure S6.** PWC analysis of the climate-plague nexus in the five countries in Western Europe in AD1347–1760. With the time series of plague outbreak adjusted by historical population figures in the corresponding country from McEvedy and Jones [1]. Panels in the left column are the PWC analysis of temperature and plague, with the effect of aridity index controlled: (A) UK, (B) France, (C) Germany, (D) Italy, and (E) Spain. Panels in the right column are the PWC analysis of aridity index and plague, with the effect of temperature controlled: (F) UK, (G) France, (H) Germany, (I) Italy, and (J) Spain. The color code for the spectrum refers to the significance of the relationship, ranging from dark red (high values) to dark blue (low values). Region with significant coherency (p < 0.05) against red noise is indicated by the black contour line. In the graph, the cone of influence indicates regions not influenced by edge effect.

**SI Text**

**SI text:**

**Data description for precipitation:**

Data of historical precipitation comes from the dataset created by Büntgen et al. [2]. The annual resolution precipitation of Europe is derived from 7284 series of European oak chronologies. Further, the anomaly of AMJ precipitation is reconstructed with respect to the 1901–2000 period. Figure S1 also shows the time series of precipitation over Europe from AD1347 to AD1760.

**Data description for historical population and its use:**

Data of historical population comes from the dataset by McEvedy and Jones [1]. The population data provides data resolution up to country scale. Any missing data is interpolated by assuming exponential growth of population in history. In Figure S4–S6, the time series of plague outbreak is divided by the corresponding population figure at the same geographical unit to control the effect of population growth in history.

Table S1. Results of Augmented Dickey-Fuller test

| Time Series | Probability |
| --- | --- |
|  | No difference |
| Plague outbreak | 0.0012 |
| Temperature | 0.0003 |
| Aridity index | 0.0000 |
| Precipitation | 0.0000 |

Table S2. Difference level and Akaike’s information criterion lag of casual linkages

| Group | Difference level | AIC lag |
| --- | --- | --- |
| Temperature 🡪 Plague outbreak | No difference | 5 |
| Aridity index 🡪 Plague outbreak | No difference | 5 |
| Precipitation 🡪 Plague outbreak | No difference | 4 |

Table S3. Simple regression result for temperature and aridity index association

|  | Coefficient | F-value | R^2^ |
| --- | --- | --- | --- |
| Temperature-aridity index association | -0.169* | 4.66 | 0.0112 |

Note: n = 414; * significant at 0.05 level

**Figure S1.** Time series of precipitation, AD1347–AD1760


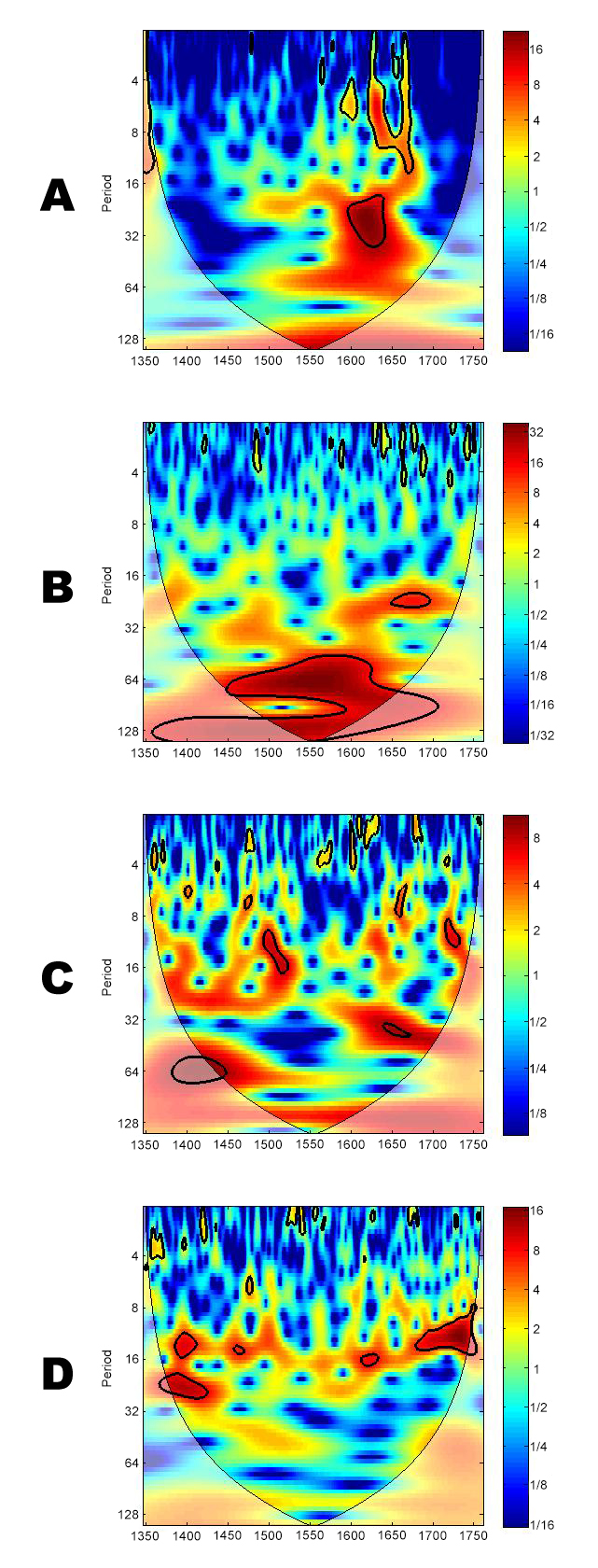


**Figure S2.** Wavelet transform of the time series of (A) plague outbreak; (B) temperature; (C) PDSI; and (D) precipitation.


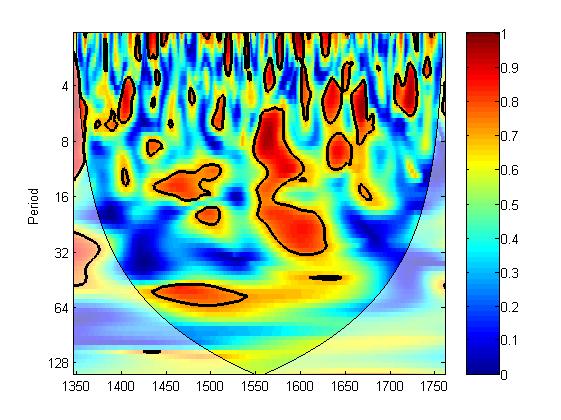


**Figure S3.** Multiple wavelet coherence (MWC) analysis, which demonstrates the synergistic effect of temperature anomalies and precipitation on plague dynamics in Europe.

Note: The result indicates a consistent coherent band between the combined effect of temperature anomalies and precipitation with plague dynamics at 64 year periodicities from AD1440–1540. Small area of Covariance can also be spotted at 8–16 year cycle and 4–32 year cycle in AD1450–1520 and AD1550–1640 respectively. By comparison to the significant region of temperature/PDSI–plague relationship (Figure 3), the temperature/precipitation–plague relationship is comparatively smaller. As suggested by Ng and Chan [3], the temperature/PDSI–plague relationship provides a better explanation to plague dynamics and its modeling.


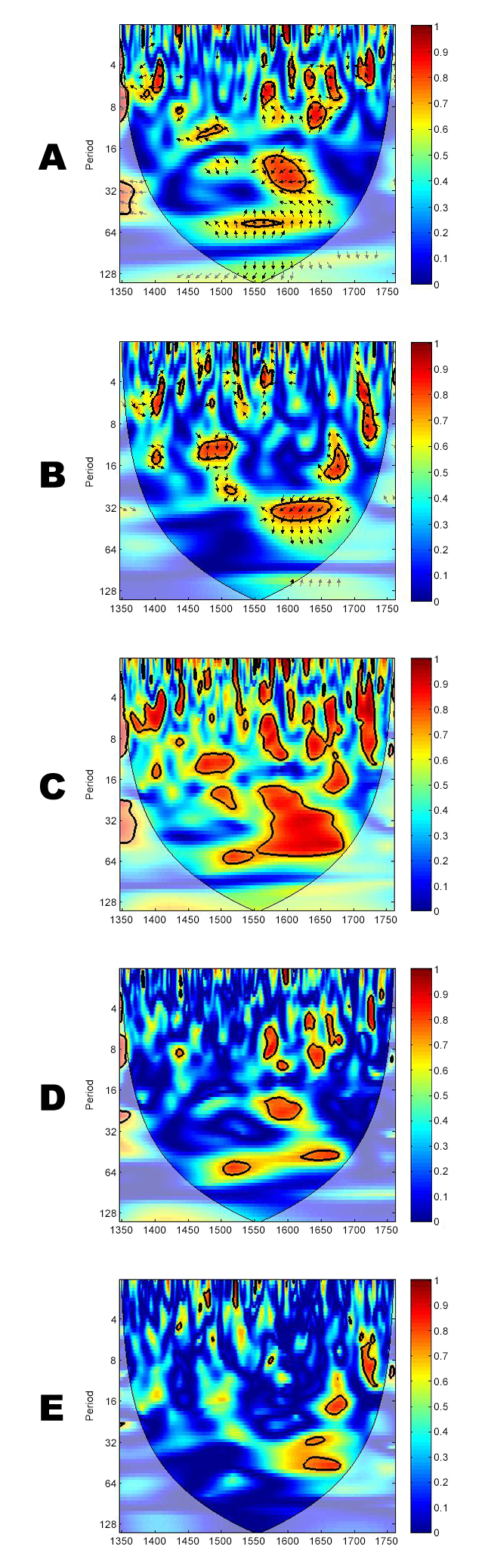


**Figure S4.** Wavelet analyses of the phase and frequency of the climate-plague nexus in Europe in AD1347–1760. With the time series of plague outbreak normalized by historical population figures from McEvedy and Jones [1]. (A) WTC analysis of temperature and plague. (B) WTC analysis of aridity index and plague. (C) MWC analysis of temperature, aridity index, and plague. (D) PWC analysis of temperature and plague, with the effect of aridity index controlled. (E) PWC analysis of aridity index and plague, with the effect of temperature controlled. The color code for the spectrum refers to the significance of the relationship, ranging from dark red (high values) to dark blue (low values). Region with significant coherency (p < 0.05) against red noise is indicated by the black contour line. In the graph, the cone of influence indicates regions not influenced by edge effect.


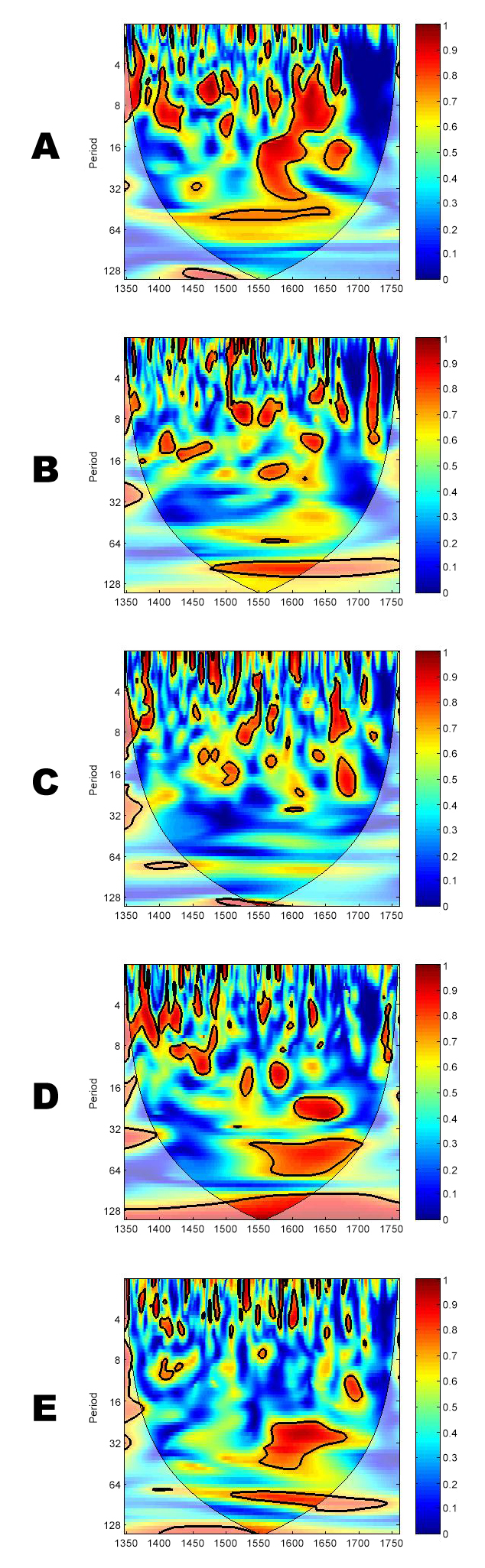


**Figure S5.** MWC analysis of the climate-plague nexus in the five countries in Western Europe in AD1347–1760. With the time series of plague outbreak adjusted by historical population figures in the corresponding country from McEvedy and Jones [1]. (A) UK, (B) France, (C) Germany, (D) Italy, and (E) Spain. The color code for the spectrum refers to the significance of the relationship, ranging from dark red (high values) to dark blue (low values). Region with significant coherency (p < 0.05) against red noise is indicated by the black contour line. In the graph, the cone of influence indicates regions not influenced by edge effect.


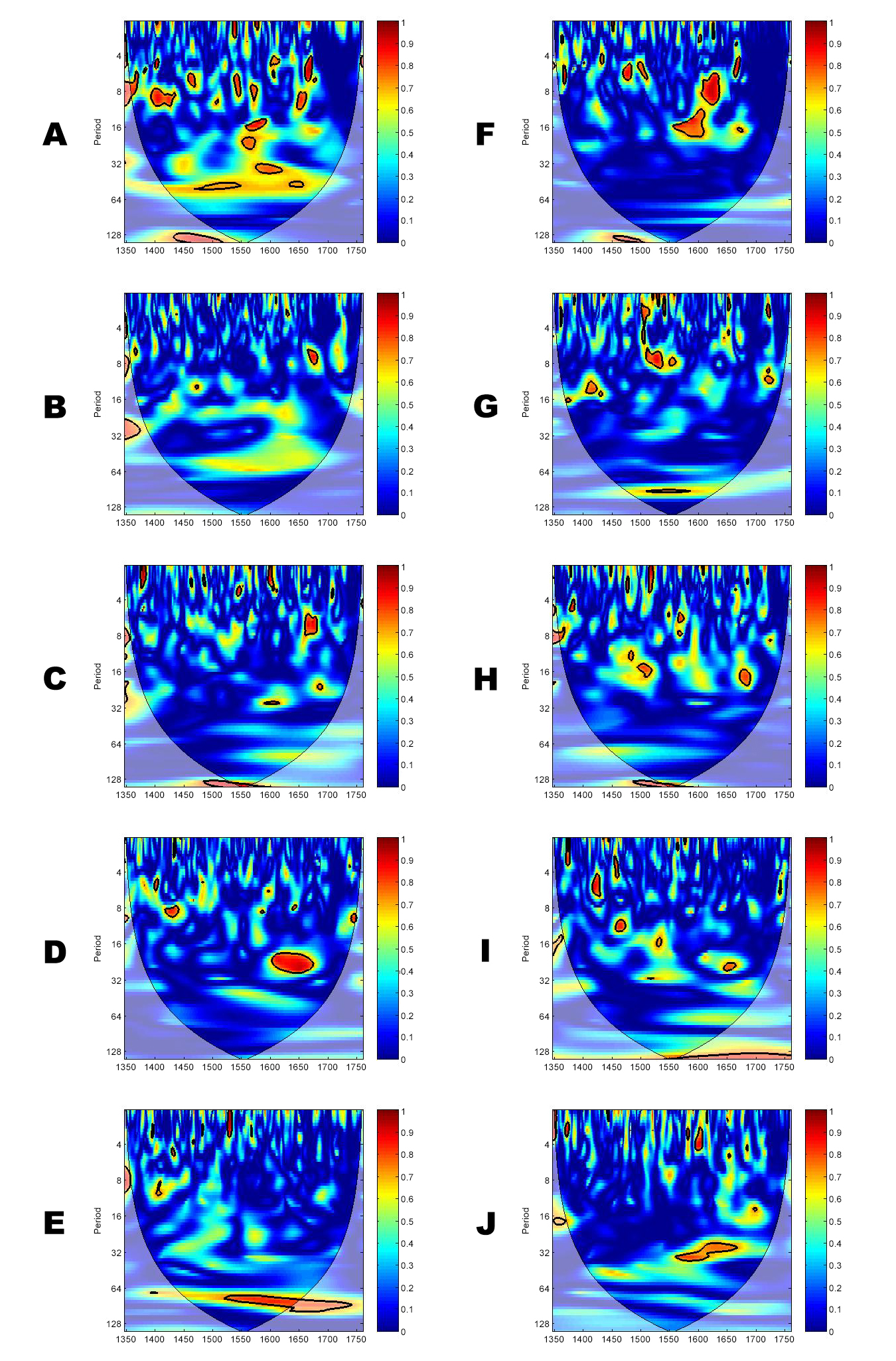


**Figure S6.** PWC analysis of the climate-plague nexus in the five countries in Western Europe in AD1347–1760. With the time series of plague outbreak adjusted by historical population figures in the corresponding country from McEvedy and Jones [1]. Panels in the left column are the PWC analysis of temperature and plague, with the effect of aridity index controlled: (A) UK, (B) France, (C) Germany, (D) Italy, and (E) Spain. Panels in the right column are the PWC analysis of aridity index and plague, with the effect of temperature controlled: (F) UK, (G) France, (H) Germany, (I) Italy, and (J) Spain. The color code for the spectrum refers to the significance of the relationship, ranging from dark red (high values) to dark blue (low values). Region with significant coherency (p < 0.05) against red noise is indicated by the black contour line. In the graph, the cone of influence indicates regions not influenced by edge effect.

Reference:

1. McEvedy C, Jones R: *Atlas of world population history.* Penguin Books Ltd, Harmondsworth, Middlesex, England.; 1978.

2. Büntgen U, Tegel W, Nicolussi K, McCormick M, Frank D, Trouet V, Kaplan JO, Herzig F, Heussner K-U, Wanner H: **2500 years of European climate variability and human susceptibility.** *Science* 2011, **331:**578-582.

3. Ng EK, Chan JC: **Geophysical applications of partial wavelet coherence and multiple wavelet coherence.** *Journal of Atmospheric and Oceanic Technology* 2012, **29:**1845-1853.
